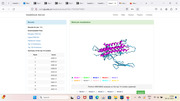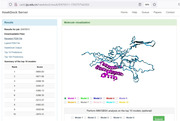# Computational Docking Analysis of APOE‐TREM2 Crosstalk with Therapeutic implications in Alzheimer’s disease (AD)

**DOI:** 10.1002/alz.084634

**Published:** 2025-01-03

**Authors:** Chand Basha SK, Janaki Ramaiah Mekala

**Affiliations:** ^1^ Koneru Lakshmaiah Education Foundation, Guntur, Andhra Pradesh India

## Abstract

**Background:**

Apolipoprotein E (APOE) and Triggering receptor expressed on myeloid cells 2 (TREM2) are the foremost genetic risk factors for late onset Alzheimer’s disease (LOAD). Recent findings suggests that crosstalk of APOE‐TREM2 has immunomodulatory effect on microglial phagocytosis, which have pathological implications in Alzheimer’s disease (AD). APOE has three isoforms: APOE (ε) 2, 3 and 4, with highest AD risk is attributed to APOE 4. R47H, a mutational variant of TREM2 increases the AD risk. The current work is done as a corroborating study, which is an extension of our work on computational investigation of TREM2 – Aβ ligands interaction.

**Method:**

The coordinates of the APOE ligands‐isoforms: 2 and 4 were imported from the PDB database. I‐TASSER server was used for the structural prediction of TREM2 and R47H TREM2 proteins. HawkDock Server was utilised for the docking of TREM2‐APOE and R47H TREM2‐ APOE.

**Result:**

The top model of TREM2 – APOE 2 docking exhibited a score of ‐ 5290.01 (Fig. 1). GBSA analysis reveals the binding free energy of the complex: ‐ 20.09 (kcal/mol). The top ranked docking model of TREM2 – APOE 4 exhibited a docking score of ‐ 5693.83 (Fig. 2) and binding free energy of the complex: ‐ 39.76 (kcal/mol). The top model of R47H TREM2 – APOE 2 docking exhibited a score of ‐ 4101.03. GBSA analysis reveals the binding free energy of the complex: ‐ 1.02 (kcal/mol). The top ranked docking model of R47H TREM2 – APOE 4 exhibited a docking score of ‐ 4969.52 and binding free energy of the complex: ‐ 10.03 (kcal/mol).

**Conclusion:**

Computational docking analysis suggests that, TREM2 exhibited highest docking scores than R47H TREM2 which signify the latter’s role in increasing AD risk. Interestingly, both TREM2 and R47H TREM2 exhibited highest docking scores against APOE 4 in comparison with APOE 2. Our computational docking analysis has therapeutic implications in AD which requires further experimental verification.